# Correlations between quantitative parameters of contrast-enhanced ultrasound and vasculogenic mimicry in murine tumor model: a novel noninvasive technique for assessment?

**DOI:** 10.1186/s12575-019-0101-5

**Published:** 2019-06-11

**Authors:** Yue-tao Zhou, Wei-wei Cai, Yue Li, Xiao Jiang, Lei Feng, Qiao-ying Zhu, Yan-ling Liu, Yu-xiao Chen, Shuang-shuang Li, Bin Du, Florian Lang, Peng-xi Wu, Li-ying Qiu

**Affiliations:** 1 Wuxi Medical School, Jiangnan University, Wuxi, 214122 Jiangsu Province, People’s Republic of China; 2 Laboratory of Tumor Pharmacology, School of Pharmaceutical Science, Jiangnan University, Wuxi, 214122 Jiangsu Province, People’s Republic of China; 3 Department of Ultrasound, Wuxi People’s Hospital, Wuxi, 214023 Jiangsu Province, People’s Republic of China; 4 Department of Physiology, Eberhard-Karls-University, Wilhelmstr. 56, D-72076 Tübingen, Germany

**Keywords:** Vasculogenic mimicry, Contrast-enhanced ultrasound, Murine tumor model, Quantitative parameter

## Abstract

**Objective:**

Vasculogenic mimicry (VM) is a novel mechanism of tumor blood supply distinct from endothelial vessel (EV). VM is associated with malignancy, invasion, metastasis, and poor prognosis. Hitherto a noninvasive method for the assessment of VM in vivo has been lacking.

**Methods:**

Contrast-enhanced ultrasound (CEUS) was performed to evaluate the quantitative parameters of tumors in mice. CD31 immunohistochemistry-Periodic Acid-Schiff double staining was conducted to identify the VM or EV in tumor tissues. Correlations between perfusion parameters and VM density was analyzed by Pearson correlation test.

**Results:**

By the 15th day after tumor inoculation, the EV and VM density was 31.15 ± 7.14 and 14.11 ± 2.99 per 200× field. The maximal intensity (IMAX) was 301.19 ± 191.56%, and the rise time (RT), time to peak (TTP) and mean transit time (mTT) were 17.38 ± 7.82 s, 20.27 ± 9.61 s and 58.09 ± 26.44 s, respectively. VM density positively correlated to RT (*r* = 0.3598, *P* = 0.0226), TTP (*r* = 0.3733, *P* = 0.0177) and mTT(*r* = 0.6483, *P* <  0.0001), whereas EV density positively correlated to IMAX (*r* = 0.4519, *P* = 0.0034). The vascular diameter of VM was substantially larger than that of EV (43.81 ± 5.88 μm vs 11.21 ± 4.13 μm).

**Conclusion:**

Three quantitative parameters related to VM were obtained and the relationships between CEUS and VM were established. CEUS might thus provide a novel noninvasive method to assess VM in vivo.

## Introduction

Tumor development is dependent on adequate vascularization. Tumor vessels not only supply nutrition and oxygen to feed tumor growth, but also provide access to systemic circulation thus facilitating tumor metastasis [1]. For many years, tumor vasculature was thought to be composed exclusively of endothelial cells (EV). In 1999, Maniotis et al. discovered a new type of microcirculatory channel in uveal melanomas, which is composed of an extracellular basement membrane and lined by aggressive tumor cells [2]. This highly patterned channel was termed vasculogenic mimicry (VM) due to its formation being independent of angiogenesis. Since then, VM has been reported in various malignant tumors, such as liver cancer, glioma, ovarian cancer, astrocytoma, and prostate cancer [3–7]. VM is associated with tumor grade, invasion and metastasis, and poor clinical prognosis [8–10]. Furthermore, the presence of VM is one of the most important factors leading to the failure of antiangiogenic therapy, even promoting tumor invasion and metastasis [11, 12]. Therefore, the identification of VM in tumors will help to grasp tumor progression and to monitor treatment.

Now VM could be identified in vitro by CD31 immunohistochemistry and Periodic Acid-Schiff (PAS) double staining [13]. But identification of VM in tumor tissues by invasive biopsy cannot reflect the global features of tumor microcirculation. It is urgent to develop a noninvasive method to evaluate VM of tumor in vivo. Ultrasound, computed tomography (CT), and magnetic resonance imaging (MRI) have been widely used to conduct vascular imaging noninvasively in animals and humans [14]. Contrast-enhanced ultrasound (CEUS) is a popular imaging technique by introducing ultrasound contrast agents into traditional medical sonography [15]. CEUS is very cost-efficient and more widely available than other molecular imaging modalities, such as MRI, PET, and SPECT. Moreover, CEUS contrast agents are in adults safer than MRI and CT radiocontrast agents. Commercially available CEUS contrast agents are gas-filled microbubbles, such as SonoVue and Optison. Microbubble size is fairly uniform, lying within a range of 1–4 μm in diameter. The introduction of microbubble contrast agents allows ultrasound to quantify perfusion at the capillary level [16, 17]. Hereby, CEUS has been used to evaluate microvessel density (MVD) in tumors and monitor response of tumors to antiangiogenic therapy [18]. The quantitative parameters of CEUS are important for the real-time evaluation of blood perfusion, such as area under the curve (AUC), rise time (RT), time to peak (TTP), maximal intensity (IMAX) and mean transit time (mTT). However, the relationships between VM and CEUS have not been well defined because recent findings indicate that tumor vasculature is heterogeneous.

In the present study, the mouse model of hepatocellular carcinoma was used to screen the quantitative parameters of CEUS related to VM. The applications of CEUS will be clinically applicable if the relationships between VM and CEUS are well defined.

## Materials and Methods

### Tumor Model

All animal care and experimental procedures described in this study were performed in accordance with the Guidelines for Animal Experiments of *Jiangnan University*. ICR mice (5–6 weeks) were purchased from Shanghai SLAC Laboratory Animal Co., Ltd. (License number: SCXK (Hu) 2013–0004).

H22 mice hepatocellular carcinoma cells were cultured with RPMI1640 (Hyclone, China) supplemented with 10% FBS (Biological Industries, Israel) under standard conditions and 1 × 10^6^ cells were implanted subcutaneously on the right flank. 25 mice were selected according to successful inoculation. Samples of tumors were rapidly harvested at 3rd, 6th, 9th, 12th, 15th days post inoculation, and fixed with 4% formaldehyde for tracking microcirculation dynamics.

Moreover, 1 × 10^6^ H22 mice hepatocellular carcinoma cells were implanted subcutaneously on the right flank. 40 mice were selected for CEUS imaging according to successful inoculation. Body weight and tumor volume were measured after inoculation.

### CEUS Imaging

By the 15th day after tumor inoculation, CEUS on mice was performed by using the Philips iU22 xMATRIX ultrasound system. Conventional ultrasound was performed with the 12 L5 probe, and CEUS was performed with the 9 L3 probe. Before CEUS imaging, mice were anesthetized and shaved.

SonoVue (Bracco, Italy) was dissolved in 5 mL physiologic saline following the instructions. A volume of 0.02 mL SonoVue contrast agent was intravenously injected manually within 20 s. CEUS imaging was started after injection and continued for 120 s. The largest cross section plane of tumor was selected for imaging. Settings and conditions were maintained during CEUS imaging.

All mice were euthanized after CEUS imaging. Samples of tumors were rapidly harvested, and fixed with 4% formaldehyde.

### CEUS Image Analysis

The CEUS clips were downloaded as DICOM format from Philips iU22 xMATRIX ultrasound system. The analysis of CEUS image was conducted by SonoLiver software (TomTec Imaging System, Germany). A region of interest (ROI) in lesion and the ROI in adjacent tissue were drawn along the perimeter of tumor based on the conventional ultrasound images. It was essential to perform motion compensation to abate the respiration noise. The time intensity curve (TIC) was generated and fitted by using a bolus kinetics model [18]. From the bolus kinetics model, four perfusion parameters were calculated for analysis, including rise time (RT), time to peak (TTP), maximal intensity (IMAX) and mean transit time (mTT). Maximal intensity was defined as the percentage ratio of intensity of ROI in lesions and that of ROI in reference at the highest during CEUS imaging. Rise time was defined as the time from 10% IMAX to 90% IMAX in ascending branch. Time to peak was defined as the time from contrast agent arrival in lesions to 100% IMAX. Mean transit time was defined as the time from 50% IMAX in ascending branch to 50% IMAX in descending branch [19, 20].

### CD31 and PAS Double Staining

Tumor tissues fixed by 4% formaldehyde were cut through the plane of maximum diameter, embedded in paraffin and cut into 5 μm sections. Immunohistochemical staining of tumor tissues was conducted using routine methods [13]. Briefly, tumor sections were deparaffinized in xylene, rehydrated through a decreasing ethanol gradient, and heated in citric acid (pH = 6.0). Endogenous peroxidases were blocked with 3% hydrogen peroxide. After the nonspecific binding sites were blocked by 10% BSA (Boster, Wuhan, China), sections were incubated overnight at 4 °C with anti-CD31 (Sangon Biotech, Shanghai, China) antibodies. In the following the sections were incubated with biotinylated goat anti-rabbit IgG (Boster, Wuhan, China). The sections were then incubated with Strept Avidin-Biotin Complex (SABC) (Boster, Wuhan, China). Immunohistochemical staining was detected by DAB (Beyotime, Nantong, China). The sections were treated with 0.5% periodic acid solution (Leagene, Beijing, China), and rinsed with distilled water. In a dark chamber, the sections were treated with Schiff solution (Leagene, Beijing, China). After distilled water rinsing, sections were counterstained with hematoxylin (Leagene, Beijing, China) followed by dehydration and coverslip mounting.

### Microvessel Counting

All microvessel counting followed procedures as previously published [21]. Briefly, the areas of highest neovascularization were found by scanning tumor sections at low magnification (× 100). After the area of highest neovascularization was identified, individual microvessel counts were made on a × 200 field. Results were expressed as the highest number of microvessels identified within any single × 200 field. At least 10 fields in chosen sections from each mouse were counted without knowledge of the previous treatment. The mean of microvessel density in 10 fields was the final outcome. Inclusions for counting: EV was positive for CD31 and PAS staining. VM was negative for CD31 staining but positive for PAS staining.

### Statistical Analysis

Data are presented as means ± standard deviations (S.D.). Relationships between quantitative parameters and EV density or VM density were analyzed by *Pearson* correlation test. *P*-values less than 0.05 were considered to be statistically significant.

## Results

### Dynamics of EV and VM Formation during Tumor Development

**Fig. 1:**
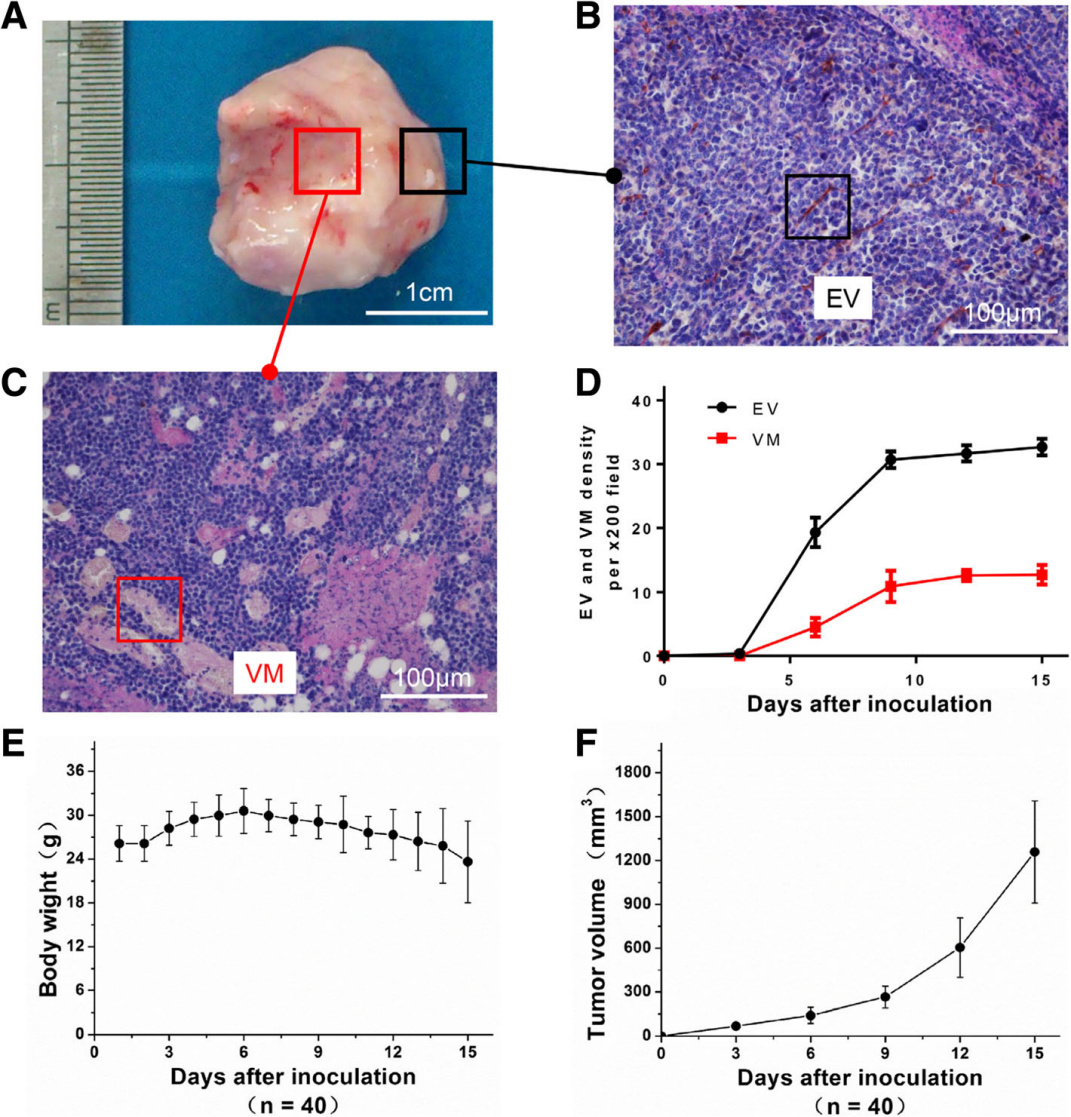
Dynamics of EV and VM formation during tumor development. **a** Representative images of H22 tumor tissues. **b**, **c** Comparison of spatial differentiation between endothelial vessels and vasculogenic mimicry under CD31 immunohistochemistry-PAS double staining (200×). **d** Endothelial vessels and vasculogenic mimicry density after tumor inoculation. **e**, **f** Dynamics of body weight change and tumor growth after tumor inoculation. Data were represented as mean ± S.D. *n* = 40

Figure 1a-c shows that EV was mainly distributed in the peripheral region of H22 tumor tissue, and VM was mainly distributed in the central region of H22 tumor tissue. There was no significant cell necrosis and inflammatory infiltration around the blood supply vessels of both tumors. In this study, the alteration of EV and VM density were obtained in H22 tumor mouse model by individual time points (Fig. 1d). Endothelial angiogenesis first appeared at the 3rd day after the inoculation of H22 tumors in mice, and the formation of VM was first observed at the 6th day after the inoculation of H22 tumor. VM appeared later than EV. At the 3rd-9th days after the inoculation of H22 tumor in mice, the density of both blood vessels increased significantly, pointing to increasing formation of EV and VM. The presence of vascular angiogenesis reflects severe hypoxia in tumor tissue. The growth of H22 tumor in mice after inoculation was slower (Fig. 1f). The increasing trend of vascular density in 9th–15th days after inoculation of H22 tumor in mice showed that the hypoxia of tumor tissue was less severe. Tumor volume increased significantly in mice 9th–15th days after H22 tumor inoculation (Fig. 1f).

### Contrast Analysis between EV and VM under Immunohistochemical Staining

**Fig. 2:**
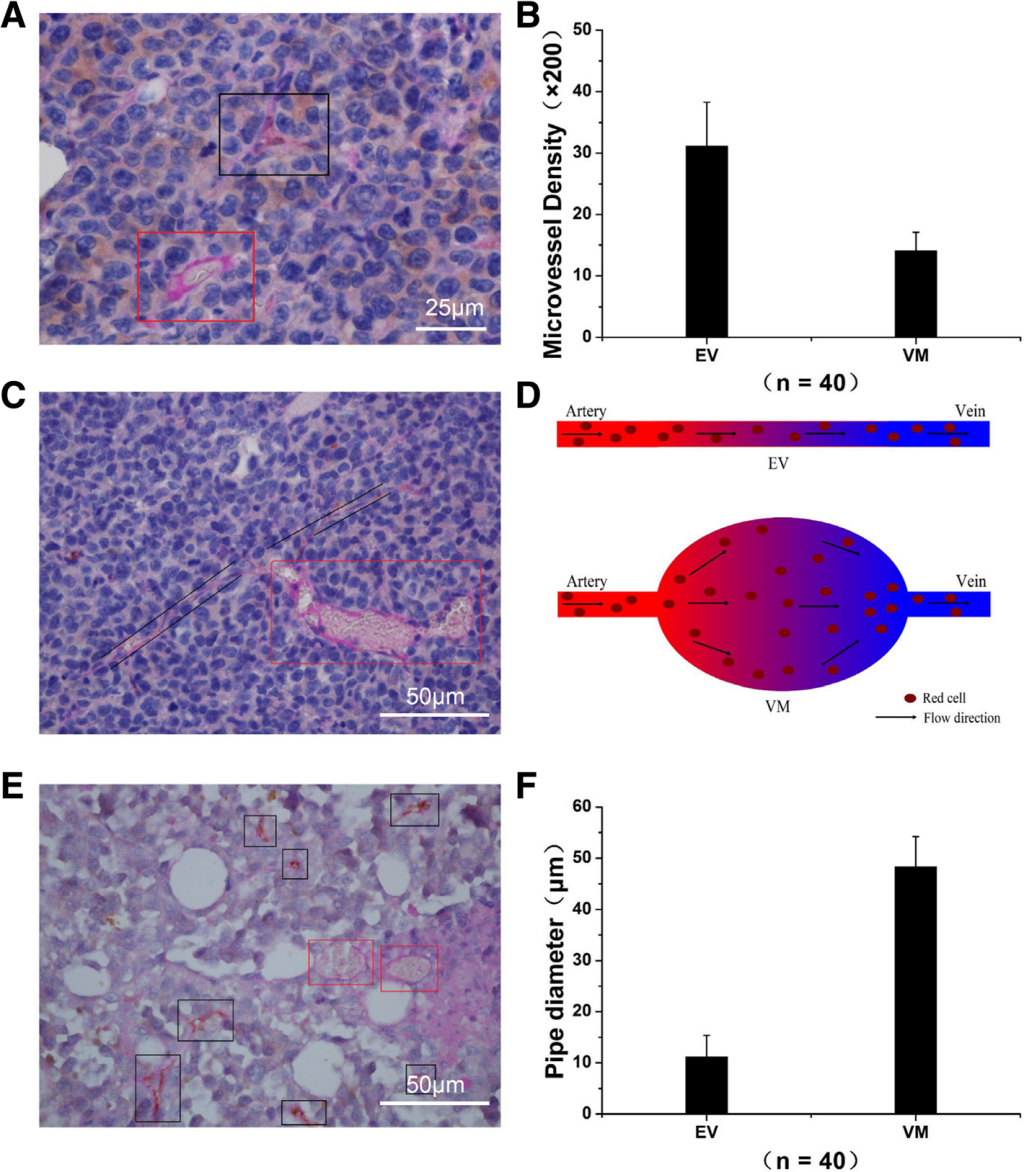
Representative pictures of CD31 immunohistochemistry-PAS double staining and contrastive analysis between EV and VM. **a** EV was positive for CD31 and PAS, whereas VM was negative for CD31 but positive for PAS. EV was labelled by black box and VM was marked by red box. **b** EV density was obviously more than VM density per 200× field. Data were represented as mean ± S.D. *n* = 40. **c** The connection between EV and VM was distinctly observed under CD31-PAS double staining (400×). EV was labelled by black box and VM was marked by red box. **d** Schematic drawing of VM and EV in two dimensional plane. When blood flows through smaller EV and bigger VM, there may be a switch between laminar flow and turbulent flow. **e** Comparison of vascular diameter between EV and VM under CD31-PAS double staining (400×). **f** The vascular diameter of VM was four times bigger than that of EV. Data were represented as mean ± S.D.

Under the CD31 and PAS double staining, EV was positive for CD31 and PAS, whereas VM was negative for CD31 but positive for PAS (Fig. 2a). The EV and VM density in tumors was 31.15 ± 7.14 and 14.11 ± 2.99 per 200× field, respectively (Fig. 2b). In other words, the EV density was significantly higher than that of VM.

Moreover, the vascular diameter of VM (labelled by red box) was larger than that of EV (labelled by black box) (Fig. 2c and e). The vascular diameters of EV and VM were 11.21 ± 4.13 μm and 48.31 ± 5.88 μm, respectively (Fig. 2f).

### Relationship between EV or VM Density and Perfusion Parameters

By the 15th day after tumor inoculation, IMAX was 301.19 ± 191.56%, and RT, TTP and mTT were 17.38 ± 7.82 s, 20.27 ± 9.61 s and 58.09 ± 26.44 s, respectively.

**Fig. 3:**
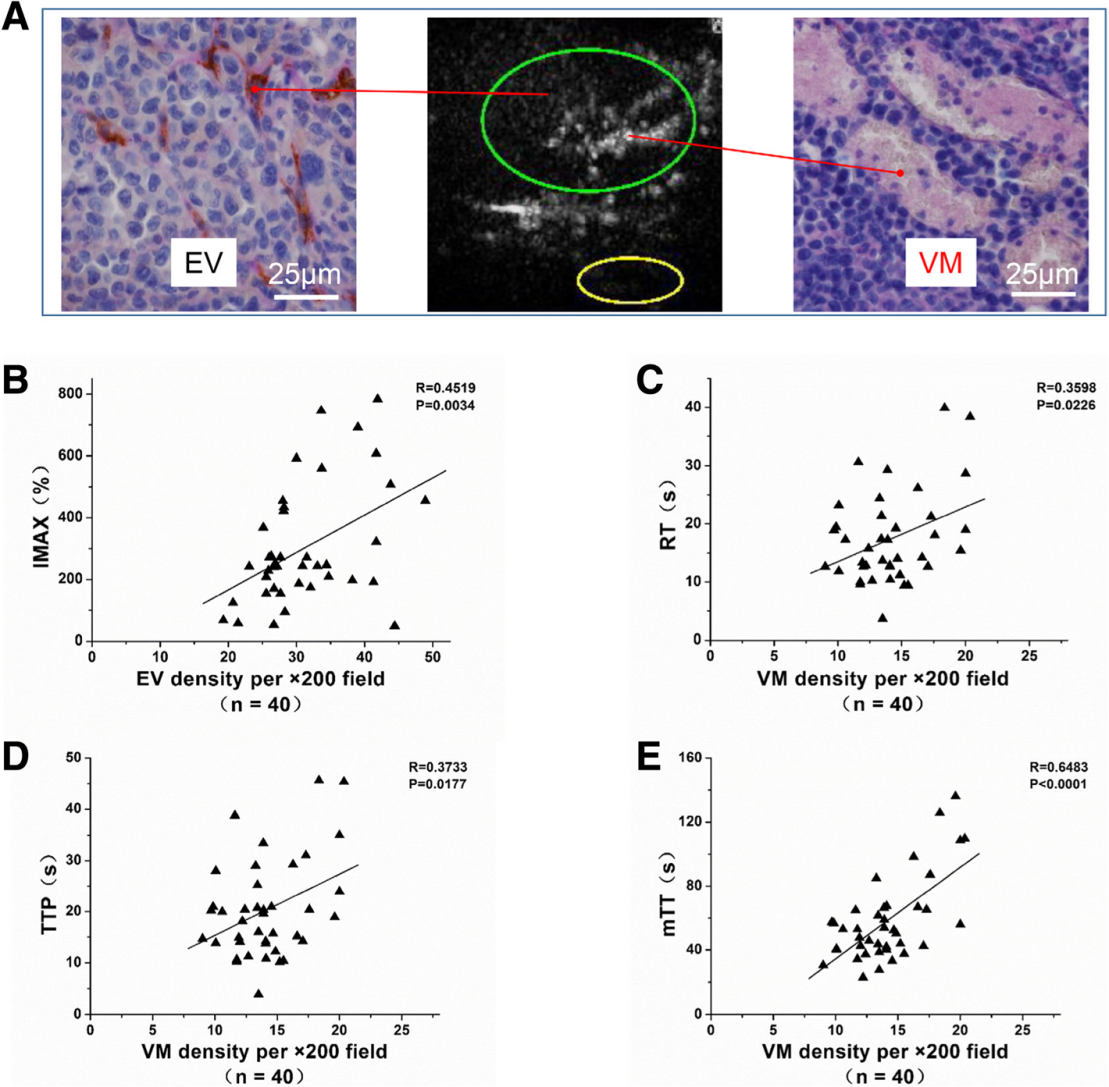
Pearson correlation test between EV or VM density and quantitive parameters of CEUS. **a** A representative CEUS image along with the corresponding CD31-PAS double staining image. **b** EV density positively correlated to the maximal intensity (IMAX) (*r* = 0.4519, *P* = 0.0034). *n* = 40. **c** VM density positively correlated to the rise time (RT) (*r* = 0.3598, *P* = 0.0226). *n* = 40. **d** VM density positively correlated to the time to peak (TTP) (*r* = 0.3733, *P* = 0.0177). n = 40. **e** VM density positively correlated to the mean transit time (mTT) (*r* = 0.6483, *P* < 0.0001). *n* = 40

There was a positive correlation between EV density and IMAX (*r* = 0.4519, *P* = 0.0034) (Fig. 3a & Tab. 1). Furthermore, there were positive correlations between VM density and RT (*r* = 0.3598, *P* = 0.0226), TTP (*r* = 0.3733, *P* = 0.0177) and mTT(*r* = 0.6483, *P* <  0.0001) (Fig. 3b, c, d & Tab. 2).

**Table 1:** Correlation between EV density and quantitive parameters of CEUS at time intensity curve

	EV density and IMAX	EV density and RT	EV density and TTP	EV density and mTT
*r*	0.4519	−0.1125	−0.0559	− 0.1219
*P*	0.0034	0.4896	0.7319	0.4535

*P*-values less than 0.05 were considered to be statistically significant. *n* = 40

**Table 2:** Correlation between VM density and quantitive parameters of CEUS at time intensity curve

	VM density and IMAX	VM density and RT	VM density and TTP	VM density and mTT
*r*	0.0178	0.3598	0.3733	0.6483
*P*	0.9130	0.0226	0.0177	< 0.0001

*P*-values less than 0.05 were considered to be statistically significant. *n* = 40

## Discussion

In recent years, various antiangiogenic drugs have passed phase II and III of clinical trials, such as Bevacizumab, Sorafenib and Cetuximab [22–24]. However, the survival benefits of antiangiogenic drugs are relatively modest. Alarmingly, anti-angiogenesis drugs even promote tumor progression, invasion and metastatic formation in some cases [25–28].

For almost 30 years, endothelial vessels (EV) have been considered the only blood supply channel in tumors. Interestingly, the VM formation differs from angiogenesis and is independent of endothelial cells. Preclinical studies have reported that antiangiogenic drugs had no effect on VM formation [29]. VM might be one of the most important factors leading to the failure of antiangiogenic therapy. Furthermore, the presence of VM is associated with tumor grade, invasion and metastasis, and poor clinical prognosis [8–10]. Obviously, VM is one of the most important factors related to tumor progression and therapy. However, there is not a noninvasive method being used to assess VM.

Use of CEUS is rapidly increasing. The most exciting advancement for ultrasound in the past 2 decades has been the introduction of microbubble contrast agents. Microbubble contrast agents are purely intravascular, providing the most accurate evaluation of enhancement [30]. A current area of prime interest for CEUS is that of evaluating microvessel density (MVD) in tumors and monitoring response of tumors to antiangiogenic therapy [18]. Correlations between CEUS quantitative parameters and microvessel density have been shown. For example, MVD has been reported to be positively correlated with IMAX and AUC [18]. Unpaired arteries negatively correlated with RT and TTP, and positively correlated with IMAX [19].

Tumor vasculature was thought to be composed exclusively by endothelial vessels (EV). In other words, EV density was mistaken to be MVD, whereas the actual MVD is mean density between EV and VM in tumors. The previous studies merely explored the relationships between EV density and quantitative parameters. The relationships between VM density and perfusion parameters of CEUS have not been well defined.

In the present study, EV density positively correlated with IMAX, which was consistent with the previous studies. IMAX was defined as the percentage ratio of intensity of ROI in lesions and ROI in reference at the highest during CEUS imaging [20]. The density of blood supply channels determined the situation of blood perfusion in tumors. Thus, the intensity of contrast agents increased with the increase in EV or VM density.

Importantly, we found that VM density was positively correlated with RT, TTP and mTT. RT and TTP reflect the arterial vascular resistance of tumors. mTT reflects the venous vascular resistance of tumor tissues. In principle, RT, TTP and mTT should decrease with the increase of microvessel density. However, according to the present study TTP and mTT increased in parallel to VM density. This interesting observation might be explained by the complex microcirculation system of tumors. Previous studies reported that (i) EV formation predominantly occurred on the periphery, whereas VM was predominantly localized in the central area [31], and (ii) significant differences in morphological structure, pipe regularity and pipe diameter occur between VM and EV [32]. Moreover, the pipe diameter of VM was four times larger than that of EV (Fig. 2f). These findings indicated there was hemadostenosis or hemangiectasis in the tumor microcirculation system. According to Poiseuille’ s Law, the viscous resistance is a linear function of viscosity, vessel length, and the fourth power of vessel radius. VM may lower the blood flow velocity in tumors. Especially, when blood flows through smaller EV and larger VM, there may be a switch between laminar flow (in EV) and turbulent flow (in VM) (Fig. 2d). Turbulent flow also lowers the blood flow velocity. Therefore, VM might lower the flow velocity of contrast agents in tumor during CEUS imaging, eventually prolonging TTP and mTT (Fig. 3).

There is a possible effect of CEUS itself on altering the vascular dynamics of tumors. Prior studies indicate that ultrasound inhibited tumor growth by disrupting tumor perfusion when the imaging duration and imaging frequency reached a certain degree. [33]. In the present study, we optimized all of these live imaging procedures to minimize the effects of CEUS on vascular dynamics, such as operating by specialist physicians, and shortening imaging duration.

There are also some limitations in the present study. First, the area under the curve (AUC), one of the parameters associated to MVD, was not included in this study due to the version difference of SonoLiver software. Second, it was difficult to ensure the immunohistological slice being identical with the CEUS imaging plane. This is a commonly known limitation when comparing histology and imaging. Third, there are obvious differences between mice models and human cancer. These restrictions further underline the need to perform prospective research to improve the quality of data and to verify the clinical applicability.

In conclusion, the relationships between CEUS and VM were established. VM is associated with cancer progression, treatment evaluation and clinical prognosis. Identification of VM in vivo will help to grasp tumor progression and to design therapeutic strategies. CEUS is one of widely used imaging modalities for lesion characterization, and its applications are mainly based on the relationship between blood perfusion and lesion. But this blood perfusion reflects the whole microcirculation and not exclusively EV or VM in tumors. CEUS will be applicable to clinical use if the relationships between VM and CEUS are well defined. In the present study, we have constructed the relationships between VM and CEUS in a murine tumor model. Next we will validate the clinical applicability by a retrospective research on the relationship between CEUS data and pathologic report of patients. The present study provides a novel noninvasive method to assess VM in vivo.

## Data Availability

All data obtained in this study can be acquired from the author according to reasonable requirements.

## Abbreviations

CEUS: Contrast-enhanced ultrasound
EV: Endothelial vessel
IMAX: Maximal intensity
mTT: Mean transit time
MVD: Microvessel density
PAS: Periodic Acid-Schiff
RT: Rise time
TTP: Time to peak
VM: Vasculogenic mimicry
